# Serum immune indicators including IgM, IgA, and IgG levels and quality of life of patients with gastrointestinal tumours during chemotherapy

**DOI:** 10.5937/jomb0-56137

**Published:** 2025-07-04

**Authors:** Mengchao Wan, Yunlong Wang, Lin Zeng, Zhiyong Zhou, Weirong Yao

**Affiliations:** 1 Jiangxi Provincial People's Hospital, The First Affiliated Hospital of Nanchang Medical College Nanchang, Department of Clinic, Jiangxi, China; 2 Jiangxi Provincial People's Hospital, The First Affiliated Hospital of Nanchang Medical College, Department of Oncology, Nanchang, Jiangxi, China

**Keywords:** gastrointestinal tumours, chemotherapy, dietetics nursing intervention, immune function, nutritional status, quality of life, IgM, IgA, IgG, gastrointestinalni tumori, hemioterapija, dijetetska intervencija u nezi, imunološka funkcija, nutritivni status, kvalitet života, IgM, IgA, IgG

## Abstract

**Background:**

Patients with gastrointestinal tumours often experience malnutrition and compromised immune function during chemotherapy, leading to a significant decline in quality of life. This study aimed to evaluate the effects of dietary nursing interventions on the nutritional status, immune function, and quality of life in patients undergoing chemotherapy.

**Methods:**

A total of 100 patients with gastrointestinal tumours receiving chemotherapy from January 2023 to June 2024 were randomly divided into two groups: a control group (CG) and a study group (SG). Both groups received conventional nursing interventions, but the SG also received dietary nursing interventions focused on personalized nutrition, dietary habits, and gastrointestinal health. Nutritional status was assessed using body mass index (BMI), haemoglobin (HGB), serum albumin (ALB), and oral mucosal cell apoptosis rate. Immune function was evaluated by measuring immunoglobulin M (IgM), immunoglobulin A (IgA), and immunoglobulin G (IgG) levels. Quality of life was measured using the GQOL-74 scale.

**Results:**

After the nursing interventions, the SG showed significant improvements in BMI, HGB, ALB, and oral mucosal cell apoptosis rate compared to the CG (P<0.05). Additionally, IgM, IgA, and IgG levels were significantly higher in the SG (P<0.05). Quality of life scores, including physical, social, psychological, and material life, also improved significantly in the SG compared to the CG (P<0.05).

**Conclusions:**

Dietary nursing interventions significantly improved the nutritional status, immune function, and quality of life of patients with gastrointestinal tumours undergoing chemotherapy. These findings underscore the importance of incorporating dietary care into nursing practices for cancer patients to support their overall well-being and treatment outcomes.

## Abstract (sr)

**Uvod:**

Pacijenti sa gastrointestinalnim tumorima često doživljavaju malnutriciju i oslabljen imunitet tokom hemioterapije, što dovodi do značajnog pada kvaliteta života. Ova studija je imala za cilj da proceni efekte dijetetskih intervencija u nezi na nutritivni status, imunološku funkciju i kvalitet života kod ovih pacijenata koji primaju hemioterapiju.

**Metode:**

Ukupno 100 pacijenata sa gastrointestinalnim tumorima koji su primali hemioterapiju od januara 2023. do juna 2024. godine nasumično je podeljeno u dve grupe: kontrolnu grupu (CG) i studijsku grupu (SG). Obe grupe su imale standardne medicinske intervencije u nezi, dok je SG dodatno dobijala dijetetske intervencije fokusi rane na personalizovanu ishranu, prehrambene navike i zdravlje gastrointestinalnog sistema. Nutritivni status je pro cenjivan pomoću indeksa telesne mase (BMI), nivoa hemoglobina (HGB), serumskog albumina (ALB) i stope apoptoze oralnih mukoznih ćelija. Imunološka funkcija je evaluirana merenjem nivoa imunoglobulina M (IgM), imunoglobulina A (IgA) i imunoglobulina G (IgG). Kvalitet života je procenjen korišćenjem skale GQOL-74.

**Rezultati:**

Nakon sprovedenih intervencija u vezi sa negom, SG je pokazala značajna poboljšanja u BMI, HGB, ALB i stopi apoptoze oralnih mukoznih ćelija u poređenju sa CG (P<0,05). Pored toga, nivoi IgM, IgA i IgG su bili značajno vi{i u SG (P 0,05). Rezultati skale kvaliteta života, uključujući fizički, društveni, psihološki i materijalni aspekt života, takođe su pokazali značajna poboljšanja u SG u poređenju sa CG (P<0,05).

**Zaključak:**

Dijetetske intervencije u nezi značajno poboljšavaju nutritivni status, imunološku funkciju i kvalitet života pacijenata sa gastrointestinalnim tumorima tokom hemioterapije. Ovi nalazi naglašavaju značaj integracije dijetetske nege u kliničku praksu za podršku op{tem blagostanju i ishodima lečenja pacijenata obolelih od raka.

## Introduction

Gastrointestinal tumours, encompassing malignancies such as oesophagal, colon, and gastric cancers, are among the most prevalent and lethal forms of cancer worldwide, contributing significantly to cancer-related morbidity and mortality [1]. These tumours often lead to a range of debilitating symptoms such as abdominal pain, dysphagia, and anorexia, which severely impact a patient’s nutritional intake and overall health [2] [3]. Treating gastrointestinal cancers typically involves chemotherapy, a primary therapeutic approach to eradicating tumour cells. However, chemotherapy is associated with various side effects, including nausea, vomiting, and mucositis, which further exacerbate nutritional deficiencies and negatively affect the patient’s quality of life [4] [5] [6]. Consequently, maintaining adequate nutrition and immune function during chemotherapy is critical to improving patient outcomes.

Malnutrition is a common and serious concern for patients undergoing chemotherapy, especially those with gastrointestinal tumours, as the treatment can cause significant alterations in appetite, digestion, and nutrient absorption [7] [8]. These patients are frequently at risk of malnutrition due to decreased food intake and increased metabolic demands resulting from the catabolic effects of chemotherapy. As a result, many experience weight loss, muscle wasting, and a decline in immune function, which in turn compromises their ability to tolerate chemotherapy and increases the risk of infection and treatment complications. Therefore, nutritional support, specifically dietary nursing interventions, plays a crucial role in managing these challenges and improving the overall well-being of cancer patients [9] [10].

Recent studies have highlighted the benefits of incorporating dietary interventions into nursing care for cancer patients, particularly those undergoing chemotherapy. These interventions ensure adequate nutrition by addressing deficiencies and promoting optimal metabolic function. Such interventions often involve personalized dietary plans that balance macronutrients, enhance digestion, and alleviate chemotherapy-induced gastrointestinal discomfort [11] [12]. Additionally, improving nutritional status has enhanced immune function, as proper nutrient intake supports the immune system’s ability to combat infections and maintain cellular integrity [13] [14].

The present study aims to evaluate the impact of dietary nursing interventions on the nutritional status, immune function, and quality of life of patients with gastrointestinal tumours undergoing chemotherapy. Specifically, we focus on serum immune indicators, including immunoglobulins (IgM, IgA, and IgG), and overall quality of life, encompassing physical, social, psychological, and material life functioning. By comparing these outcomes between patients receiving conventional care and those receiving dietary nursing interventions, we seek to demonstrate the importance of incorporating dietary support into the standard care protocol for this patient population.

## Materials and methods

### General data

The 100 patients with gastrointestinal tumours undergoing chemotherapy in our hospital from January 2023 to June 2024 received selection and division into a control group (CG) and a study group (SG) according to different nursing methods, with 50 cases each. CG: 28 males and 22 females; mean age was (53.7±10.3) years old; disease course was (2.20±0.20) years; tumour types: 14 cases of oesophagal carcinoma, 14 cases of colon cancer, and 22 cases of gastric cancer. SG: 26 males and 24 females; mean age was (53.2±10.7) years old; disease course was (2.12±0.25) years; tumour types: 11 cases of oesophagal carcinoma, 14 cases of colon cancer, and 25 cases of gastric cancer. This research received approval from our hospital’s ethics committee.

### Inclusion and exclusion criteria

Inclusion criteria: 1) The diagnostic results all met diagnostic criteria for gastrointestinal tumours provided by the current Chinese Medical Association; 2) patients and their family members signed informed consent; 3) with sufficient compliance and could cooperate with research. Exclusion criteria: 1) With other diseases or mental disorders; 2) patients and their family members did not agree to participate in this research; 3) lack of compliance and inability to actively cooperate with research.

### Methods

The CG received conventional chemotherapy nursing intervention. Nursing staff should provide psychological counselling, health education, conventional dietary guidance, medication guidance, and other nursing interventions to patients. Nursing staff should introduce to patients the causes of their own diseases and aspects they need to pay attention to in daily life. Meanwhile, nursing staff should guide patients to take drugs correctly, ensuring that patients can take drugs scientifically and reasonably. Patients may experience negative emotions such as anxiety and unease due to the impact of their illness. Nursing staff should communicate with patients and provide psychological counselling for patients to improve their compliance.

The SG received dietary nursing based on conventional nursing in CG. 1) Nursing staff should understand and master patients’ daily dietary habits and develop comprehensive and detailed dietary plans based on patients’ condition and dietary habits. Nursing staff should guide patients in choosing healthy and nutritious foods and dietary habits to ensure their nutritional balance and adequacy. 2) Nursing staff should inform patients and their family members in detail and accurately about dietary precautions and correct dietary habits, enhance the digestive effect of patients after eating, and guide patients to eat more vegetables, grains, and legumes, avoid spicy, greasy, and cold foods, thereby mitigating irritation to the digestive tract, and preventing adverse reactions. Nursing staff should guide patients to take vitamin-rich foods, drink plenty of water, consume garlic sprouts, etc., to enhance gastrointestinal motility. 3) Nursing staff should conduct a risk assessment of patients’ dietary conditions and take preventive measures against potential adverse dietary conditions. Nursing staff should take anti-emetic and appetite-enhancing drugs for patients during the nursing process to improve their dietary deficiencies. If patients experience vomiting or other symptoms, nursing staff should intervene with fluid replacement and guide patients to have more meals a day but less food at each.

### Observation indicators

(1) Nutritional status: The nutritional indicators, including body mass index (BMI), oral mucosal cell apoptosis rate, haemoglobin (HGB), and serum albumin (ALB) levels between both groups received comparison. The normal HGB level is 110–165 g/L, and the normal serum ALB level is 35–50 g/L. The lower the oral mucosa cell apoptosis rate, the more severe the malnutrition.

(2) Immune function: The immune indicators, including immunoglobulin M (IgM), immunoglobulin A (IgA), and immunoglobulin G (IgG) levels before and after nursing between both groups received comparison.

(3) Quality of life: The quality of life before and after nursing between both groups received comparison. The GQOL-74 scale [15] assessed patients’ physical, social, psychological, and material lives. The maximum score is 100 points; the higher the score, the higher the patients’ quality of life.

### Statistical analysis

The SPSS 27.0 software received an application to process data. Count data received representation as [n (%)], followed by χ^2^ test for intergroup comparisons. Quantitative data that conform to normal distribution received expression as (x̄±s), followed by a t-test for intergroup comparisons. P<0.05 indicated a statistically significant difference.

## Results

### Comparison of general data

There was no statistical significance in the general data between the two groups (P>0.05; Table 1), suggesting that the groups were comparable in gender, age, disease course, and tumour type before the intervention. This ensured that any observed outcome differences could be attributed to the intervention rather than baseline characteristics.

**Table 1:** General data in both groups. CG, control group; SG, study group

Groups	N	Gender [n (%)]		Age<br>(years)	Disease course<br>(years)	Tumour type [n (%)]		
		Male	Female			Esophageal<br>carcinoma	Colon<br>cancer	Gastric<br>cancer
CG	50	28 (56.0)	22 (44.0)	53.70±10.30	2.20±0.20	14 (28.0)	14 (28.0)	22 (44.0)
SG	50	26 (52.0)	24 (48.0)	53.20±10.70	2.12±0.25	11 (22.0)	14 (28.0)	25 (50.0)
χ^2^/t		0.322		0.22	0.057	1.103		
P		0.57		0.827	0.955	0.576		

### Comparison of nutritional indicators

**Figure 1:**
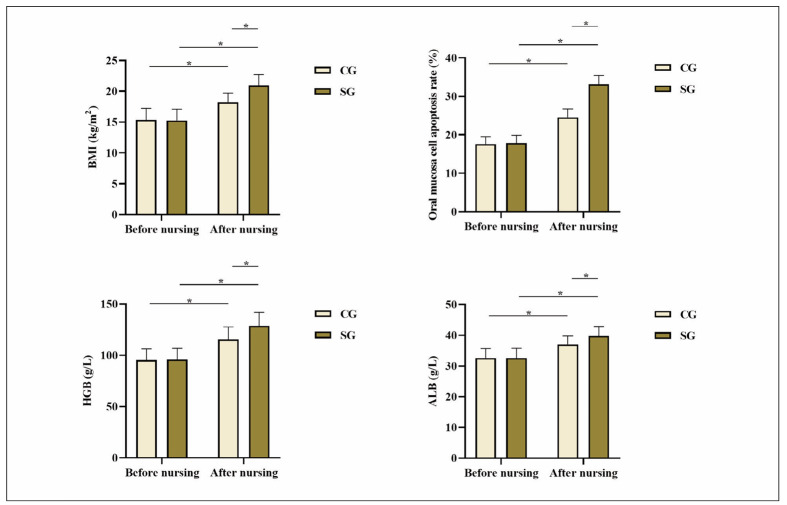
Nutritional indicators in both groups. *P<0.05. CG, control group; SG, study group

Before nursing, no statistical significance in BMI, oral mucosa cell apoptosis rate, HGB level, and serum ALB level was exhibited between both groups (P>0.05). After nursing, BMI, oral mucosa cell apoptosis rate, HGB level, and serum ALB level in both groups exhibited elevation relative to those before nursing, and SG exhibited elevation relative to CG, indicating statistical significance (P<0.05; Figure 1).

### Comparison of immune indicators

**Figure 2:**
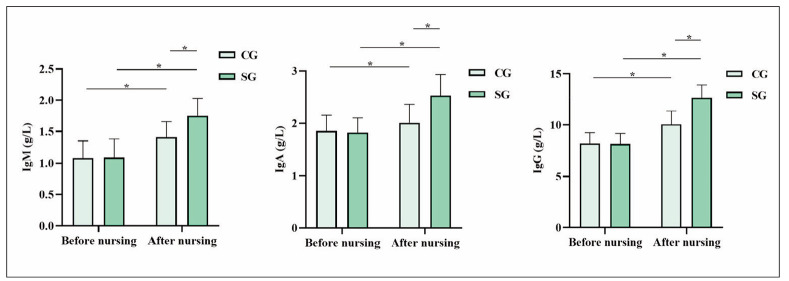
Immune indicators in both groups. *P<0.05. CG, control group; SG, study group

Before nursing, no statistical significance in IgM, IgA, and IgG levels exhibited between both groups (P>0.05). After nursing, IgM, IgA, and IgG levels in both groups exhibited elevation relative to those before nursing, and SG exhibited elevation relative to CG, indicating statistical significance (P<0.05; Figure 2).

### Comparison of quality of life

**Figure 3:**
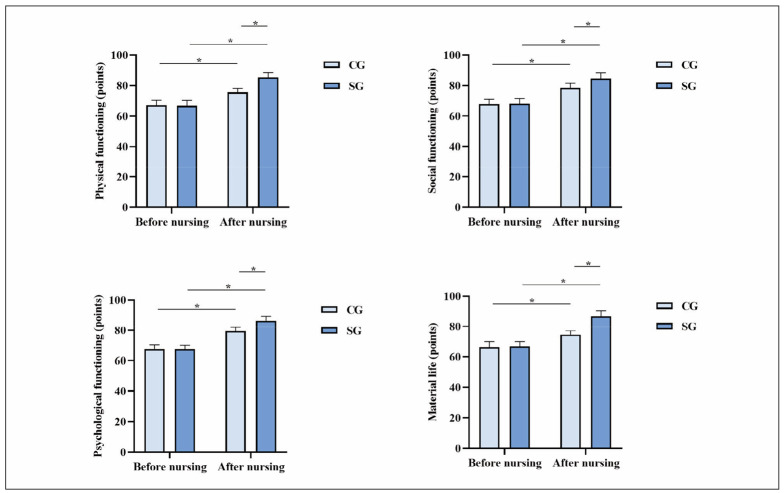
Quality of life in both groups. *P<0.05. CG, control group; SG, study group

Before the intervention, the two groups had no significant differences in physical, social, psychological, or material life functioning scores (P>0.05). After the nursing interventions, both groups showed improvements in all quality-of-life domains. However, the SG exhibited significantly higher scores than the CG in all aspects of quality of life, including physical, social, psychological, and material life functioning (P<0.05; Figure 3), indicating the positive impact of dietary nursing intervention on overall well-being.

## Discussion

Gastrointestinal tumours are common diseases that cause eating and digestive dysfunction in patients due to the location of the attack [16] [17]. Cancer patients often experience changes in taste and smell, with a decrease in the threshold for bitterness and an increase in the threshold for sweetness, and usually present manifestations such as loss of appetite, anorexia, unpleasant odours, abdominal distension, constipation, nausea, and vomiting, all of which can affect patients’ appetite and lead to malnutrition [18]. Chemotherapy, one of the primary treatment methods for malignant tumours, has received wide application in clinical practice. Chemotherapy can cause varying degrees of toxic reactions in patients while exerting an anti-tumour role against tumours [19] [20]. Most anti-tumour drugs applied in clinical practice can cause gastrointestinal reactions, and nausea and vomiting are the most common, which can lead to chemotherapy termination or change, causing malnutrition [21]. The major reason is that drugs stimulate medullary chemical vomiting centres to cause nausea and vomiting reflexively. Patients’ metabolic rate accelerates during the chemotherapy process, requiring more energy consumption; moreover, there may be varying degrees of adverse reactions during chemotherapy [22]. Thus, it is necessary to provide scientific dietary nursing interventions for patients with gastrointestinal tumours during chemotherapy.

Patients with gastrointestinal tumours often experience weight loss and malnutrition [23], and their nutritional needs should be taken seriously, including intake of protein, carbohydrates, and vitamins [24] [25]. Protein is the major nutrient for patients, and the body’s daily protein requirement is usually 0.8–1.0 g/kg, while the daily protein requirement of patients is 1.5 g/kg, which should account for 20%–25% of total calories. During chemotherapy, due to reduced food intake caused by vomiting, the body utilizes stored raw materials, and energy required by cancer cells majorly relies on metabolic process of converting glucose to lactate; if intake is insufficient, protein consumed receives conversion to glucose. Additionally, during chemotherapy, effects of drugs on normal tissue cells enable patients to provide sufficient protein and calories, otherwise it can cause muscle catabolism and rapid weight loss. At this time, for chemotherapy patients, to maintain their basal metabolism, it is necessary to elevate protein to over 2 g/kg per day. Carbohydrates majorly participate in endogenous metabolism of proteins and prevent catabolism of tissue, which are the major source of calories, with a daily requirement of 350–500 g/kg. Only by consuming sufficient carbohydrates can protein utilization and storage be enhanced. During chemotherapy, patients should also ensure supplementation of vitamins. Vitamin C, an antioxidant, exerts a protective role against occurrence and development of tumours, can block synthesis of nitrosamines in the body, and exerts an adjuvant therapeutic impact on tumours.

This research developed targeted patient dietary plans based on their daily dietary habits, ensuring balanced and sufficient nutrition and avoiding unhealthy dietary habits and food. After nursing, BMI, oral mucosa cell apoptosis rate, HGB level, and serum ALB level in SG exhibited elevation relative to those in CG, indicating that dietary nursing intervention effectively improved the nutritional status of patients. The improvement in immune function observed in the SG, reflected by the significant elevation in IgM, IgA, and IgG levels, further underscores the critical role of dietary nursing interventions in supporting the immune system of patients with gastrointestinal tumours undergoing chemotherapy. Chemotherapy-induced immune suppression is a significant concern, as it compromises the body’s ability to fight infections and recover from treatment. By providing tailored dietary interventions that meet the patient’s specific nutritional needs, it is possible to mitigate the negative impacts of chemotherapy on immune function. The elevated immunoglobulin levels in the SG group suggest that dietary support addresses the immediate nutritional deficiencies and contributes to a stronger immune defence, which is crucial for improving patient outcomes and enhancing the overall quality of life. These findings align with existing literature highlighting nutritional support’s positive effects on cancer patients’ immune function. Clinical research has demonstrated that providing nutritional support to patients as early as possible can facilitate the absorption of nutrients in the gastrointestinal tract and positively enhance immune function [26] [27]. Herein, after nursing, IgM, IgA, and IgG levels in SG exhibited elevation relative to those in CG, indicating that dietary nursing intervention effectively improved the immune function of patients. If the body cannot obtain sufficient nutrition for a long time, the operation of various systems, organs, spinal cord hematopoiesis, etc., will deteriorate, and the brain will experience symptoms such as dizziness and coma due to lack of nutrition, resulting in a decline in overall function; furthermore, consumption of nutrients by tumours can lead to excessive daily burden on the body, which can accelerate death. Herein, physical functioning scores, social functioning scores, psychological functioning scores, and material life scores in SG exhibited elevation relative to those in CG, indicating that dietary nursing intervention effectively improved patients’ quality of life. It is because dietary nursing interventions can adjust patients’ emotions, behaviours, etc., enrich patients’ cognition with various knowledge and increase their daily diet frequency, ensuring nutritional supply and metabolic balance; adequate nutrition in the body can prevent malignant tumour cells from invading other healthy tissues, and the body can obtain necessary nutrients and increase the number of healthy cells, better fighting against diseases.

In conclusion, dietary nursing interventions for patients with gastrointestinal tumours undergoing chemotherapy effectively improve nutritional status, enhance immune function, and elevate quality of life. This approach represents a significant and beneficial nursing strategy that should be widely adopted and incorporated into the standard care practices for patients with gastrointestinal tumours undergoing chemotherapy.

## Dodatak

### Acknowledgements

Not applicable.

### Funding

Not applicable.

### Ethics approval and consent to participate

This study was approved by the Ethics Committee of Jiangxi Provincial People’s Hospital, The First Affiliated Hospital of Nanchang Medical College and was conducted in compliance with the guidelines of the Declaration of Helsinki. The written informed consent was obtained from each patient.

### Availability of data and materials

The data and materials used to support the findings of this study are available from the corresponding author.

### Authors’ contributions

Mengchao Wan and Yunlong Wang designed the study. Mengchao Wan, Lin Zeng, Zhiyong Zhou, and Weirong Yao performed the data analysis and visualization. Mengchao Wan and Yunlong Wang drafted the manuscript and revised the manuscript. All authors read and approved the final manuscript.

### Conflict of interest statement

All the authors declare that they have no conflict of interest in this work.

### List of abbreviations

CG, control group;<br>SG, study group;<br>BMI, body mass index;<br>HGB, haemoglobin;<br>ALB albumin;<br>IgM, immunoglobulin M;<br>IgA, immunoglobulin A;<br>IgG, immuno globulin G.
